# Dr. J. N. Willard

**Published:** 1914-12

**Authors:** 


**Dr. J. N. Willard,** Bellevue 1875, of Utica, died in Syracuse, September 25, aged 70.

